# Development of Screening Tools for the Interpretation of Chemical Biomonitoring Data

**DOI:** 10.1155/2012/941082

**Published:** 2012-02-16

**Authors:** Richard A. Becker, Sean M. Hays, Steven Robison, Lesa L. Aylward

**Affiliations:** ^1^Regulatory and Technical Affairs Department, American Chemistry Council, Washington, DC 20002, USA; ^2^Summit Toxicology, LLP, Lyons, CO 80540, USA; ^3^Central Product Safety, Procter & Gamble, Cincinnati, OH 45253, USA; ^4^Summit Toxicology, LLP, Falls Church, VA 22044, USA

## Abstract

Evaluation of a larger number of chemicals in commerce from the perspective of potential human health risk has become a focus of attention in North America and Europe. Screening-level chemical risk assessment evaluations consider both exposure and hazard. Exposures are increasingly being evaluated through biomonitoring studies in humans. Interpreting human biomonitoring results requires comparison to toxicity guidance values. However, conventional chemical-specific risk assessments result in identification of toxicity-based exposure guidance values such as tolerable daily intakes (TDIs) as applied doses that cannot directly be used to evaluate exposure information provided by biomonitoring data in a health risk context. This paper describes a variety of approaches for development of screening-level exposure guidance values with translation from an external dose to a biomarker concentration framework for interpreting biomonitoring data in a risk context. Applications of tools and concepts including biomonitoring equivalents (BEs), the threshold of toxicologic concern (TTC), and generic toxicokinetic and physiologically based toxicokinetic models are described. These approaches employ varying levels of existing chemical-specific data, chemical class-specific assessments, and generic modeling tools in response to varying levels of available data in order to allow assessment and prioritization of chemical exposures for refined assessment in a risk management context.

## 1. Introduction

Recognition of the large numbers of chemicals in commerce and increased focus on evaluation of these chemicals from the perspective of potential human health risk has become a focus of attention in North America and Europe. These efforts are devoted not only to evaluation of “new” chemicals but also to an examination of existing chemical substances. These efforts include those under the Health Canada Chemicals Management Plan, the European Registration, Evaluation, Authorisation and Restriction of Chemicals (REACh), the High Production Volume (HPV) Challenge Program, and the US Environmental Protection Agency's (US EPA) Chemical Assessment and Management Program (ChAMP) initiatives. Chemical evaluation is also being discussed as part of potential improvements to the US Toxic Substances Control Act. Because of the large number of chemicals involved and the need for efficient processes that assure focus on substances which could pose the greatest health concerns, tiered approaches that begin with conservative risk-based screening-level assumptions and proceed to more refined data-intensive approaches have been recommended for these types of efforts [1, 2]. 

Chemical risk assessment evaluations consider both exposure and hazard, and a tiered set of approaches employing various levels of data for screening-level assessments is often recommended [1, 3, 4]. Exposure screening considers chemical uses, identifies potential exposure media or pathways, and invokes conservative assumptions in the estimation of potential daily exposure rates. Hazard evaluation includes the identification of established tolerable exposure levels (e.g., reference doses or tolerable daily intakes [RfDs or TDIs]). In the absence of such established guidance values, robust no observed adverse effect levels (NOAELs) or benchmark doses (BMDs) can be used as a point of departure (POD) and adjustment factors for extrapolation applied (as necessary), and margins of safety (MOS) can then be calculated for risk-based screening. Finally, in the absence of robust toxicological data, a generic screening approach such as that developed under the threshold of toxicological concern (TTC) framework [5–7] for setting conservative tolerable intake rates has been widely used.

In this paper, we explore approaches for using chemical biomonitoring data in risk assessment evaluation of chemicals. As with external exposure-based assessments, exposure assessments based on biomonitoring data require health- or risk-based benchmarks for evaluation of biomarker data. However, because biomarker data is typically expressed in units of biomarker concentration (e.g., *μ*g/L urine) and risk-based benchmarks are typically expressed in units of applied dose (mg/kg-day), direct comparison cannot be made. Two approaches are possible: (1) the biomarker can be back calculated to an applied dose (reverse dosimetry; see, e.g., [8]), or (2) the benchmark can be forward calculated to a corresponding biomarker concentration for use as a screening value (forward dosimetry; see Hays et al. [9]). 

**Figure 1:**
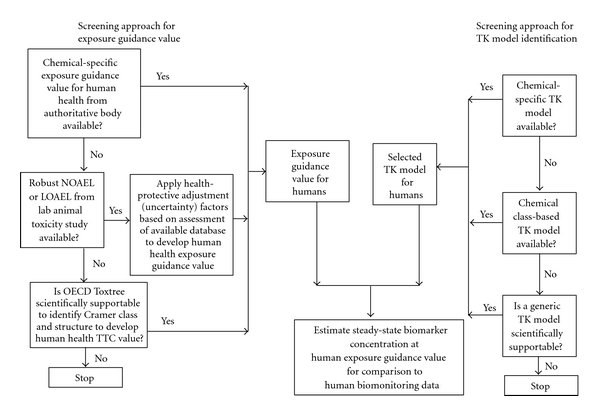
Flowchart showing approaches for development of screening values for assessment of biomonitoring data for chemicals with varying levels of available data on both hazard and toxicokinetics.

Dosimetry calculations, whether forward or reverse, require the use of pharmacokinetic data and modeling and assumptions regarding exposure patterns. This paper describes methods for interpreting human biomonitoring data in a risk context, illustrating the use of the forward dosimetry biomonitoring equivalents approach for five scenarios. The first three are applicable to substances for which toxicokinetics are well understood but that have different levels of toxicity data: (1) substances with established government risk assessments, (2) substances with sufficient toxicity datasets but as of yet no government-generated (or -vetted) risk assessment, and (3) substances amenable to the generic screening TTC approach for setting conservative tolerable intake rates. These latter two approaches are needed because, for many chemicals in common use today, there may not be authoritative, government-conducted, or “approved” chemical-specific risk assessment-based exposure guidance values available. The additional scenarios addressed in this paper include (4) the absence of chemical-specific toxicokinetic data or models, and (5) the absence of both toxicity-based guidance values and toxicokinetic data. The framework of the cases and approaches described here is summarized in Figure 1 and discussed in detail below.

## 2. Using Risk Assessment Methods to Interpret Human Biomonitoring Results

Human biomonitoring, in which chemicals or their metabolites, are measured in biological media such as blood or urine, has become a powerful tool in the assessment of chemical exposures in the general population and in studies of targeted populations [10, 11]. Human biomonitoring data provide a reflection of integrated exposure from multiple pathways and routes in terms of internal, biologically relevant dose. In situations in which exposures to a chemical potentially occur through multiple or ill-defined exposure routes or pathways, well-designed and conducted human biomonitoring studies can provide robust and reliable exposure data that can complement and refine or replace external exposure estimation based on more indirect approaches and generic assumptions. Biomonitoring can be particularly useful in cases where widespread population exposure is possible (e.g., residues of agricultural chemicals, food packaging constituents, consumer product ingredients, etc.). Biomonitoring can also be used as an accessory tool in evaluation of exposure to chemical ingredients in consumer products in targeted, controlled exposure studies (see below for example with triclosan).

### 2.1. Biomonitoring Equivalents Based on Substances with Established Government Risk Assessments and Established Toxicokinetics

Screening criteria for determining the health significance of human biomonitoring results would ideally be based on robust datasets relating potential adverse effects to biomarker concentrations in human populations (see, e.g., the US Centers for Disease Control and Prevention (CDC) blood lead level of concern; see http://www.cdc.gov/nceh/lead/). However, data to support such assessments exist for only a few environmental chemicals because this approach requires establishment of causality in epidemiological studies and a robust understanding of human dose response. Thus, in an alternative approach, the concept of biomonitoring equivalents (BEs) has been developed, and guidelines for the derivation and communication of these values have been published [9, 12, 13].

In conventional risk assessment, concentrations in environmental media are used with specific contact scenarios to derive an estimate of external dose (mg/kg-day), and this is then compared to an external dose health-based guidance value, such as an ADI, RfD or TDI (mg/kg-day). In the initial screening-level evaluation, estimated exposure rates are compared to hazard- or risk-based benchmarks to assess whether more refined evaluations are required. When an RfD, or TDI or analogous screening value such as a TTC is available, the screening-level exposure estimate is compared directly to that value to assess whether exposure rates above that value are anticipated. If a NOAEL or other POD is used as the benchmark, then adjustment factors (AFs) (synonymous with uncertainty factors or safety factors) are generally used to extrapolate from animal toxicity to humans (default 10x) and to account for human variability (default 10x). Depending upon the database and quality of studies, additional AFs may be used [14]. If a toxicity database is not robust, use of an additional database uncertainty factor should be considered. Once the screening level health-based exposure guidance value has been determined, then a margin of safety (MOS) can be calculated by comparing this to the estimated daily dose rate (*D*):

(1) $$\text{MOS}=\frac{\left(\text{POD}/\text{AFs}\right)}{D}.$$

MOS values below 1 indicate that exposures exceed the screening level health-based exposure guidance value. If screening approaches have been used in the exposure or hazard assessment process, further refinement in those assessments may be warranted. Such refinements to provide greater certainty of potential hazards and exposures may include generation of product-specific exposure data for chemical uses with higher estimated exposure rates, conducting specific toxicity studies to address database deficiencies, or other exposure or hazard characterization refinements. Results of refined assessments can be used to identify the need for, and useful focus of, potential risk management strategies.

In the biomonitoring equivalent approach for interpreting biomonitoring exposure data (internal dose concentrations) in a risk assessment context, external dose health-based guidance values are translated to estimates of corresponding steady-state biomarker concentrations. A biomonitoring equivalent (BE) is defined as the concentration or range of concentrations of a chemical or its metabolites in a biological medium (blood, urine, or other medium) that is consistent with an existing health-based exposure guidance value such as a reference dose (RfD) or tolerable or acceptable daily intake (TDI or ADI) [12]. BEs are intended to be used as screening tools to provide an assessment of which chemical biomarkers are present at levels well below, near, or at or above concentrations that are consistent with existing risk assessments and exposure guidance values, and thus can provide an evaluation of relative priority for risk assessment followup. BEs provide a translational tool allowing application of the foundational risk assessment paradigm to the evaluation of exposure information provided by biomonitoring data. Development of BE values requires an underlying exposure guidance value (such as an RfD or TDI) as well as sufficient understanding of pharmacokinetics of the chemical in humans or key laboratory species. BEs are similar in concept to the HBM-I assessment values derived by the German human biomonitoring council (reviewed in Angerer et al. [15]). For interpreting human biomonitoring data in a risk context, the margin of safety (MOS) approach is used

(2) $$\text{MOS}=\frac{\text{BE}}{\left[\text{Biomarker}\right]}.$$

When the MOS value is 1 or greater, then the exposure to the substance is not likely to be of concern.

BE values have been derived for approximately 80 chemicals in a variety of chemical classes (see Angerer et al. [15] for review). BE derivations have been published for persistent organic compounds including dioxins, hexachlorobenzene, and DDT and metabolites, for approximately 40 volatile organic compounds, for several phthalates and phenols including di-2(ethylhexyl)phthalate, bisphenol A, and triclosan, for selected pyrethroid pesticides, and for selected brominated flame retardant compounds. For many of these chemicals, multiple BE values have been derived corresponding to different available risk assessment exposure guidance value (e.g., EPA RfDs versus TDI values derived by the European Food Safety Authority [EFSA]). For these chemicals, screening level assessments of population biomonitoring data can be made by comparison of the data to the BE value corresponding to the risk assessment exposure guidance value deemed most appropriate.

### 2.2. Risk-Based Interpretation of Biomonitoring Based on Substances with Sufficient Toxicity Datasets but No Government-Generated (or Approved) Risk Assessment

Establishing comprehensive, risk assessment-based exposure guidance values such as RfDs or TDIs is a resource-intensive effort that may take several years to complete for substances with extensive datasets. In many cases, substantial toxicological data exist for chemicals, but no formal risk assessment-based exposure guidance values such as an RfD or TDI have yet been established by a government agency. Further, some existing risk-assessment based values may now be outdated, based on the availability of newer, more relevant hazard or exposure data. Thus, for many chemicals in common use today, there may not be authoritative, government-conducted or -approved chemical-specific risk assessment-based exposure guidance values available. In the absence of such established guidance values, robust no observed adverse effect levels or benchmark doses based on a review of available datasets can be used as a point of departure, and by use of appropriate AFs, screening level health-based exposure guidance values can be derived. If appropriate pharmacokinetic data are available, these screening level health-based exposure guidance values can be translated to corresponding internal biomarker concentrations and used to assess human biomonitoring data in a parallel fashion. A MOS based on comparison of the biomonitoring data to the biomarker concentration level consistent with the screening level health-based exposure guidance value can then be calculated.

An example of this approach has been presented by Aylward and Hays [16] for the flame retardant hexabromocyclododecane (HBCD). Although a substantial database of toxicity data for both standard and endocrine-sensitive endpoints is available, no exposure guidance values have been established. Both Health Canada and the European Union have conducted provisional or draft risk assessments in which sensitive PODs were identified [17, 18]. Data were available on measured or estimated lipid-adjusted HBCD concentrations in experimental animals at the identified POD dose levels. Substantial data on lipid-adjusted HBCD concentrations in human serum and milk were available and tabulated. Comparison of those data to the biomarker concentrations in the animal studies at the PODs showed margins of exposure (MOEs) in excess of 5,000 for general population exposures to HBCD [16]. In this case, a MOE comparison was made, which is analogous to the MOS approach, except with the MOE, AFs are not used, and comparison is made directly to the POD.

A similar MOE approach was incorporated as part of a risk assessment for triclosan conducted by the European Commission Scientific Committee on Consumer Products (ECSCCP) [19]. In this case, serum concentrations of triclosan were measured throughout the course of a chronic animal bioassay selected by the ECSCCP as the basis for establishment of a TDI. Thus, serum concentrations in rats corresponding to the NOAEL dosing regimen were directly available from the toxicological database. In contrast to HBCD, in which general population exposures are incidental and due to trace levels of HBCD released into the environment, triclosan is added intentionally as an antibacterial agent to a variety of directly applied and used personal care products such as toothpaste or soap. Thus, direct consumer exposure is anticipated. The conventional risk assessment approach entails estimation of exposure levels using generic assumptions about each use scenario, contact rates, absorption, and so forth. However, because consumers may experience exposures to multiple products containing triclosan, with potential exposure via more than one route (dermal, ingestion), the conventional exposure assessment process can be cumbersome, requiring assessment of many exposure scenarios and reliance on multiple conservative, potentially compounding, exposure assumptions.

In the ECSCCP evaluation, in addition to a conventional MOE assessment based on estimated external doses from use of multiple products compared to an animal NOAEL, a biomarker-based assessment was also conducted. Peak serum levels were measured in volunteers using multiple triclosan-containing products (toothpaste, deodorant stick, and hand soap) and compared to the serum levels at the NOAEL in rats in the chronic bioassay. The conventional assessment based on estimated external doses resulted in an MOE of approximately 380 compared to the administered dose rates in rats at the NOAEL. The corresponding assessment based on comparison of human serum levels to serum levels measured in the animal bioassay at the NOAEL resulted in an MOE of approximately 940. This result confirms (1) that the approach based on estimated external exposures incorporates conservative assumptions and (2) the practical utility of risk-based screening using biomonitoring data.

The triclosan example illustrates the value of including measurements of blood biomarker concentrations in toxicological assays, as recommended by Barton et al. [20] and Saghir et al. [21]. Biomarker concentrations, and in particular blood or serum concentrations of chemicals, provide a reflection of biologically relevant absorbed dose and tissue concentrations. Comparison of biomarker concentrations in humans under real-world product use scenarios to the corresponding biomarker concentrations in laboratory animals under bioassay conditions at the POD potentially reduces uncertainties associated with reliance on estimated external exposure doses in the process of safety assessment of products.

Interpreting human biomonitoring data in a risk context for substances that lack comprehensive, health-based exposure guidance values is challenging.

Programs such as Health Canada's Chemicals Management Plan, the European Union (EU) Registration, Evaluation, Authorisation, and Restriction of Chemical (REACh), and the US Toxic Substances Control Act (HPV Challenge Program and ChAMP), while they may be lacking health-based exposure guidance values, can often provide sufficient data to support this screening-level approach.

For example, under the High Production Volume (HPV) Challenge Program (http://www.epa.gov/chemrtk/index.htm) which is now substantially complete, toxicity data and other relevant information on approximately 2,200 chemicals produced or imported into the US, in quantities >1,000,000 lbs./year, has been submitted to EPA to enable screening based on the OECD's SIDS paradigm. This data, which covers about 90–95% by volume of chemicals in commerce in the US, is publicly available and was evaluated by EPA, under the Chemical Assessment and Management Program (ChAMP) initiative, to derive screening-level hazard characterizations, and then, for a subset of these, a screening-level risk-based prioritization. From its initiation in 2007 to 2009, when it was superseded, EPA's ChAMP developed 786 hazard characterizations and 220 risk-based prioritizations [22, 23].

For each of these substances, the hazard characterizations generated by EPA provide a concise assessment of the toxicity data and include delineation of LOAELs and NOAELs for effects on (1) major organ systems (from both acute and repeated exposures), (2) the developing organism in utero, (3) reproduction, and (4) the fidelity of DNA (http://www.epa.gov/champ/). The LOAELS or NOAELs (as appropriate) for these substances can be readily accessed from EPA's HPVIS online database (http://www.epa.gov/hpv/hpvis/index.html) and used for deriving a POD. These values are typically expressed as applied doses in mg/kg-day. AFs for toxicodynamics can then be applied to derive a screening level health-based exposure guidance value, which is also in units of applied dose (mg/kg-bw/day). Then, by using chemical-specific toxicokinetic data or models (CSTK), a biomarker concentration level typically in units of concentration in blood or urine consistent with this screening level health-based exposure guidance values can be developed. Biomonitoring results can then be interpreted in a risk context using the MOS procedure.

When using this approach, it is important to recognize that the typical AFs of 10x for extrapolating to animals to humans and 10x to account for human variability each contain both dynamic and kinetic components [24]. Thus, to use this method to interpret human biomonitoring data, when deriving the screening level health-based exposure guidance value from a NOAEL or POD based on an oral toxicity lab animal study, it is important to use in the first step only the dynamic components of the AFs (typically 2.5x or 3.16x to extrapolate from animals to humans and 3.16x to account for human variability) should be used [24, 25]. Then, in a second step, the CSTK data or model needs to be used to convert the applied dose into a concentration and in doing so, the CSTK may allow replacement of the kinetic components of the typical AFs. If both the typical 10x for extrapolating from animals to humans and the 10x to account for human variability are applied to the lab animal toxicity NOAEL and the CSTK is also applied, “double counting” for toxicokinetics would occur.

### 2.3. Risk-Based Interpretation of Biomonitoring Based on the Thresholds of Toxicological Concern (TTC) Method

In some cases, no robust toxicological data on which to base selection of a POD are available for a chemical. In this case, the TTC approach can provide a method for selection of a provisional, conservative tolerable daily dose level based on historical data and distributions of NOAEL values (or cancer potency values) along with an appropriate uncertainty factor (or low dose linear extrapolation factor) for a wide range of compounds [5–7, 26, 27]. The threshold of toxicological concern (TTC) evolved from concepts initially developed by Frawley [28] and further refined by the US FDA as the threshold of regulation [29, 30] and was initially developed based on extrapolated risk data for carcinogens with the assumption that if the carcinogenicity endpoint was protected, all other toxicological endpoints would also be protected. These concepts were considerably expanded to include consideration of chemical structure in conjunction with toxicity data for other toxicological endpoints [5, 27]. One of the most important enhancements to the original work was the consideration of chemical structure and the addition of a decision tree linked to exposures that pose little or no health risk. The acceptable exposure levels were derived by an extensive analysis of the existing toxicology data for 730 chemicals tested for carcinogenicity (low dose risk based) and more than 600 chemicals tested for repeat dose toxicity (NOAEL based) [31].

The TTC approach provides a decision tree linking chemical structure with toxicity. Chemical characteristics are used to identify a generic, conservative tolerable daily intake rate, the TTC. The TTC approach is based on an analysis of two comprehensive databases of toxicity data: one that is relevant to genotoxic carcinogens and one that is relevant to repeat dose endpoints not predicted on an assumption of potential genotoxic carcinogenicity. These tools are used by first assessing conservatively whether or not the chemical has structural features (alerts) suggestive of the potential for carcinogenicity via a genotoxic mode of action. Chemicals with alerts for potential genotoxic carcinogenicity are subject to an exposure limit based on the distribution of potencies of historically tested carcinogens. Chemicals without alerts for genotoxicity may move further along the decision tree, and, based on their structures, be categorized into one of three classes that are associated with three different conservative tolerable intake rates, or TTCs [27]. The category-specific recommended TTC levels are considered to be conservative estimates of chronic daily intake rates that are unlikely to result in adverse effects. This is based on the analysis of the distribution of no observed adverse effect levels (NOAELS) for compounds in the three categories. These values are based on the 5th percentile NOAELS along with the application of default uncertainty factors [5–7]. Applying the TTC approach permits rapid evaluation of exposure levels to chemicals with little or no chemical-specific toxicology data to determine if exposures are sufficient to trigger concern for a potential for health risk. Exposures below the TTCs are judged to pose a very low probability of an appreciable risk to human health.

Although the approach was originally developed to support exposures to indirect food additives and later to dietary exposures, the underlying datasets are broad and, consequently, application of the TTC concept to a broader range of exposure scenarios has been considered [32–37]. Initial development and application of the TTC approach was focused on systemic exposure resulting from oral administration or exposure to compounds. More recently, the TTC approach was extended to consider systemic exposure following topical application of cosmetic products [32, 35]. There has also been the suggestion that TTC can be applied to inhalation exposure and risk assessment [33, 35–37]. It also has been proposed that the TTC can be applied to intentionally added materials found at low concentrations in food [7, 34]. Although there are broad categories of chemicals that can be evaluated using the TTC, there are certain materials that have insufficient data in the underlying toxicity datasets, have been identified as carcinogens with potencies that fall outside of the distribution, or have concerns related to bioaccumulation for the TTC to be applied. These include metals, organometals, and the polyhalogenated dioxins, furans and biphenyl derivatives [27].

Application of the TTC requires a careful evaluation of the chemical(s) under consideration and application of the decision tree to assign the chemical to the appropriate tier of the decision tree. This decision tree is outlined in several publications [27] and has been implemented as part of the OECD QSAR Toolbox (available at http://www.oecd.org/document/54/0,3746,en_2649_34379_42923638_1_1_1_1,00.html). An additional module is also available for identifying alerts for carcinogenicity that may be used as part of the weight of the evidence on whether or not to consider the chemical as a potential genotoxic carcinogen. Use of the decision tree approach offers a way to prioritize which materials need more in-depth evaluation.

The TTC methodology was developed to evaluate the potential for risk to low-level exposure to chemicals in the diet and has subsequently been applied to ingredients or contaminants in pharmaceutical and consumer products. Since biomonitoring represents a “real-world” measurement of such low-level exposure application of the TTC principles offers an approach to evaluating the measured exposures in a risk-based context. TTC values are typically expressed as applied doses, as either mg/kg-day or mg/day (for a defined population). To use a TTC value to interpret human biomonitoring data thus requires conversion to an internal dose concentration. If sufficient chemical-specific toxicokinetic data are available, the TTC could be translated into a corresponding biomarker concentration under the assumption of chronic steady-state exposure at the TTC level. As discussed above, in converting to an internal dose concentration, attention must be paid to proper application of AFs for dynamics and kinetics to avoid “double counting.” This typically will entail review of the derivation of TTC, removal of the default AF used for toxicokinetics, then applying chemical-specific toxicokinetic data or models to obtain an internal biomarker concentration level equivalent to the TTC. Biomonitoring results can then be interpreted in a risk context using the MOS procedure. This would also provide a way to identify chemicals where additional biomonitoring would add little value. For example, if a chemical was in Cramer Class 3, which has an assigned TTC of 90 *μ*g/day, and the biomonitoring data such as those from national biomonitoring programs such as the US National Health and Nutrition Examination Survey (NHANES) or the Canadian Health Measures Survey (CHMS) indicated, through reverse dosimetry estimations, that exposure levels at the 95th percentile were likely orders of magnitude less than 90 *μ*g/day, that chemical could be a candidate for removal from the biomonitoring program.

In a practical sense, for a chemical with little or no toxicological data for which the TTC approach is used to identify a screening intake level, the chemical-specific toxicokinetic data or measurements required to estimate corresponding biomarker values may not be available. In such cases, generic toxicokinetic approaches may be considered; these are discussed further below.

### 2.4. Risk-Based Interpretation of Biomonitoring in the Absence of Chemical-Specific Toxicokinetic Data or Models

For many chemicals, risk assessment-based exposure guidance values or robust POD values are available. However, little or no chemical-specific toxicokinetic data may exist because such data have not necessarily been considered to be part of the core toxicological test batteries used to assess chemical safety. For such chemicals, provisional estimates of biomarker concentrations corresponding to key benchmarks may still be possible, albeit with greater uncertainty or built-in conservatism.

One approach relies upon a read-across from other chemicals that are structurally similar or that have similar chemical and physical properties. If chemicals are closely related, data for a well-studied chemical may be used and serve as a surrogate for a structurally similar compound with fewer data. Recently criteria have been established for structural analog identification and selection [38], and this process has been validated with a set of case studies [39].

More broadly, chemicals that exhibit similar physical and chemical properties may be evaluated using a generic model applicable to that class. For example, Chiu and White [40] demonstrated the derivation and application of steady-state solutions to a generic physiologically based toxicokinetic (PBTK) model for volatile organic compounds (VOCs) in route-to-route extrapolation. The steady-state solutions require very limited chemical-specific data to implement, and such data can often be generated in vitro [41]. Aylward et al. [42] collected the required chemical-specific data from the literature as well as current risk assessment-based exposure guidance values for approximately 40 VOC compounds. They implemented the steady-state solutions to the generic PBTK model to estimate steady-state blood concentrations predicted to arise from steady-state exposures.

The resulting estimated chemical-specific blood concentrations corresponding to exposure guidance values were proposed for use as screening values for evaluation of biomonitoring data for these VOCs [42]. Across this class of compounds, variation in physical/chemical and metabolic properties resulted in estimated steady-state blood concentrations for a unit inhalation exposure (e.g., 1 mg/m^3^) that varied by approximately one order of magnitude, while those arising from a unit of oral exposure (1 mg/kg-day) varied over approximately 2 orders of magnitude [42]. Therefore, if an exposure guidance value is available for a chemical expected to have similar physical, chemical, and metabolic behavior to those included here, a range of likely steady-state blood concentrations potentially consistent with the exposure guidance value could be estimated.

Other PBTK model structures potentially applicable to a wider range of compounds have been proposed and used in a variety of contexts. Rotroff et al. [43] used in vitro methods to develop estimates of the metabolic clearance and protein binding for a series of chemicals included in the US EPA Phase I ToxCast program. These parameters were used in a generic PBTK model to relate blood or serum concentrations to corresponding steady-state external dose rates using commercially available software. Bartels et al. [44] presented initial results of a comprehensive effort to develop a generic PBTK model structure that accommodates varying levels of chemical-specific information and allows prediction of biomarker concentrations (both urinary and blood) associated with a specified exposure guidance value. On the toxicity assessment front, Louisse et al. [45] demonstrated the integration of in vitro toxicity data with toxicokinetic models to assess glycol ethers. These efforts highlight the potential utility of targeted data development including in vitro assessments of metabolism and measured or estimated chemical and physical properties in allowing development of provisional biomarker screening or assessment values based on current risk assessments. If human biomonitoring data approach or exceed these screening values, allocation of resources to development of more detailed, data-driven evaluations of toxicokinetic characteristics may be appropriate.

### 2.5. Risk-Based Interpretation of Biomonitoring in the Absence of Both Guidance Values and Toxicokinetic Data

Most of the chemicals currently being assessed in the US NHANES and the Canadian Health Measures Surveys are well-studied substances. However, even among this group of compounds, there is sometimes a lack of derived toxicity guidance values, and, more commonly, a lack of detailed chemical-specific toxicokinetic data needed to translate external exposure levels into expected corresponding biomarker concentrations as required to support development of BEs. As biomonitoring programs are expanded to include less well-studied substances, compounds that lack both comprehensive toxicity datasets, and toxicokinetic data needed for development of full BE values are likely to be included. In such cases, provisional screening assessment values may still be derived using combinations of the approaches outlined above.

**Figure 2:**
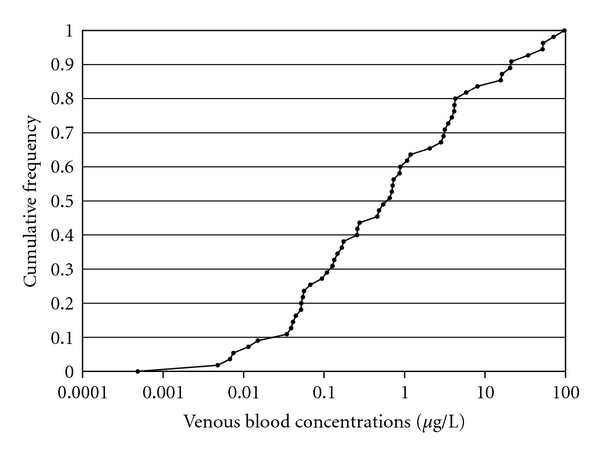
Estimated steady-state venous blood concentrations corresponding to oral and inhalation exposure guidance values (56 values) for 38 VOCs from Aylward et al. [42]. Some chemicals had both oral and inhalation exposure guidance values, while others had only one.

For example, the Aylward et al. [42] evaluation of screening BE values for assessment of VOCs could be applied to chemicals lacking both exposure guidance values and toxicokinetic data. The screening values estimated by Chiu and White [40] incorporate both the toxicokinetic behavior of the chemicals as well as the risk assessment-based tolerable exposure levels based on noncancer endpoints. The cumulative distribution of estimated screening blood concentrations for these VOCs is presented in Figure 2. The values span more than five orders of magnitude in blood concentration. If a chemical is judged to be similar in general physical, chemical, and toxicological characteristics to those included in the group evaluated by Aylward et al. [42] but lacks the information necessary for a chemical specific BE, a lower percentile of blood concentration from the distribution represented here might be selected as an initial screening value for evaluation of blood concentrations of that chemical measured in humans. This approach is conceptually similar to the TTC approach, but conducted on a biomarker concentration basis rather than an intake dose basis. 

Similarly, the TTC approach could be applied to a chemical to estimate a conservative level of tolerable external exposure, and a generic PBTK model such as that developed by Bartels et al. [44] could be used to estimate a corresponding biomarker concentration for use as a screening value.

### 2.6. Decision Tree for Screening Level, Risk-Based Interpretation of Biomonitoring Data

Figure 1 provides a general flowchart of the various approaches described here. The flowchart is conceptually similar to the tiered screening process described in a 2001 review by the Health Council of the Netherlands [46], with the added component of extension of the tiered approach to evaluation of biomonitoring data. These approaches should be applied in an iterative framework, with increasing refinement indicated when MOS values are judged to be insufficient. Use of all-generic approaches to derive provisional screening values clearly results in values that are highly uncertain, requiring the use of health-protective assumptions in the screening process. If chemicals being detected in biomonitoring surveys fall into this category of lacking both toxicological and toxicokinetic data, these chemicals may be candidates for early research to fill selected data gaps in order to refine the assessments for those chemicals.

## 3. Discussion and Conclusions

The collection and reporting of human biomonitoring data continues to grow, and the advanced analytical chemistry techniques employed can now accurately quantify substances in reasonable sample volumes of blood or urine from individuals. And while authoritative organizations have cautioned that detection does not equate to illness or injury, the absence of methods to interpret human biomonitoring in a health risk context reduces the value of these data because of the inability to prioritize among the detected chemicals on the basis of potential risk posed by the detected levels. Employing tools to interpret biomonitoring data which results in a risk assessment-based context can assist risk managers in addressing concerns about chemical exposures. It also provides a framework for determining whether additional product stewardship and/or regulatory risk management actions may be warranted.

The BE approach has proven to be useful as a screening tool to provide an assessment of which chemical biomarkers are present at levels well below, near, or at or above concentrations that are consistent with exposure guidance values derived in existing authoritative government risk assessments. As discussed here, the underlying approach developed for the BEs can also be used in cases where such authoritative risk assessments are not yet available or where robust toxicokinetic models aren't at hand. Both the NOAEL approach and the TTC method discussed here can be used to establish benchmarks that will allow screening-level evaluation of biomonitoring data. Although there are uncertainties when using such methods, by employing health protective assumptions, such as additional uncertainty factors to account for database shortcomings, the derived Points of Departure from the NOAEL and TTC approaches can be used with a reasonable degree of confidence that they are health protective.

As with any method used for chemical exposure assessment, the quality and representativeness of the biomonitoring data must be considered in the process of interpreting the data. While a complete discussion of the factors relevant to evaluation of biomonitoring data is outside the scope of this paper, some of these factors include the stability and specificity of the biomarker and the representativeness of the sampling frame used to generate the data. Similarly, the robustness and reliability of the toxicokinetic models and data used to translate affect the confidence in the derived Biomonitoring Equivalents (discussed in Hays et al. [9]).

The methods described here represent a range of approaches that can be applied depending on the level of chemical-specific information available. Obviously, as the level of chemical-specific data decreases and reliance on generic assumptions increases, the uncertainty associated with the derived screening values increases. If human biomonitoring data approach or exceed these screening values, allocation of resources to development of more detailed, data-driven evaluations may be appropriate in order to inform risk managers. In such cases, an iterative approach to development and application of human biomonitoring assessment values is appropriate. Such an approach allows for and takes advantage of targeted data development. Such data may include in vitro assessments of metabolism, measured or estimated chemical and physical properties, or in vivo toxicokinetics and metabolism studies to refine provisional toxicokinetic estimates.

## Abbreviations

ADI: Acceptable daily intake
AF: Adjustment factor
BE: Biomonitoring equivalent
BMD: Benchmark dose
CDC: Centers for Disease Control and Prevention
ChAMP: Chemical Assessment and Management Program
CHMS: Canadian Health Measures Survey
CSTK: Chemical-specific toxicokinetic
ECSCCP: European Commission Scientific Committee on Consumer Products
EFSA: European Food Safety Authority
EPA: Environmental Protection Agency
HBCD: Hexabromocyclododecane
HBM-I: Human biomonitoring value-I
HPV: High production volume
MOE: Margin of exposure
MOS: Margin of safety
NHANES: National Health and Nutrition Examination Survey
NOAEL: No observed adverse effect level
PBTK: Physiologically based toxicokinetic
POD: Point of departure
REACh: Registration, evaluation, authorisation and restriction of chemicals
RfD: Reference dose
TDI: Tolerable daily intake
TTC: Threshold of toxicological concern
VOC: Volatile organic chemical.
